# Editorial on Special Issue “Spine Imaging: Novel Image Acquisition Techniques and Analysis Tools”

**DOI:** 10.3390/diagnostics12061361

**Published:** 2022-06-01

**Authors:** Nico Sollmann, Thomas Baum

**Affiliations:** 1 Department of Diagnostic and Interventional Radiology, University Hospital Ulm, Albert-Einstein-Allee 23, 89081 Ulm, Germany; 2 Department of Diagnostic and Interventional Neuroradiology, School of Medicine, Klinikum Rechts der Isar, Technical University of Munich, Ismaninger Str. 22, 81675 Munich, Germany; thomas.baum@tum.de; 3 TUM-Neuroimaging Center, Klinikum Rechts der Isar, Technical University of Munich, 81675 Munich, Germany

Imaging of the spine, including radiography, computed tomography (CT), and magnetic resonance imaging (MRI), is frequently performed in clinical routine. This relates to the high prevalence of disorders affecting the spine that can be captured and characterized by imaging, including degenerative spine diseases and specifically osteoporosis and associated vertebral fractures (VFs) [1,2,3], traumatic injuries [4,5,6], and oncologic conditions such as primary bone tumors, metastatic spread, or multiple myeloma [7,8,9,10]. Contemporary imaging thus plays a key role in diagnosis, therapy monitoring, and computer-assisted planning of surgical interventions along the spine.

In the light of recent developments in CT and MRI scanner hardware and software, together with advancements in image data analysis based on artificial intelligence (AI), this Special Issue entitled “Spine Imaging: Novel Image Acquisition Techniques and Analysis Tools” focused on (I) advances in image acquisition, including radiography, dual energy X-ray absorptiometry (DXA), multi-detector CT (MDCT), and MRI; (II) novel post-processing methods for image reconstruction or advanced analysis pipelines; and (III) advances in automated image segmentation and diagnostic support tools (particularly AI-based approaches).

The non-invasive quantification of fat is possible with chemical shift encoding-based water–fat MRI (CSE-MRI), which enables the extraction of the proton density fat fraction (PDFF) as a fundamental tissue property calculated as the ratio of density of mobile protons from fat (triglycerides) and the total density of protons from mobile triglycerides and mobile water [11]. With regard to the musculoskeletal system, the CSE-MRI-based PDFF has been primarily used for fat quantification of the bone marrow (BM) in vertebral bodies in osteoporosis or for vertebral body endplate lesions [1,12]. For the latter, the discrimination between Modic-type endplate lesions as a degenerative entity and infectious diseases including spondylodiscitis is a common issue for clinical imaging, which has been addressed in the work by Schmeel et al. [13]. Specifically, the authors used the PDFF derived from CSE-MRI (3D six-echo modified Dixon sequence at 1.5 or 3 Tesla) for distinguishing Modic type 1 endplate lesions from infectious spondylitis, with histopathology or clinical and imaging follow-up examinations as the diagnostic reference standards [13]. The intra-vertebral PDFF and a PDFF ratio (i.e., vertebral endplate PDFF divided by normal vertebrae PDFF) were calculated within edematous BM lesions, and measurements were then compared between patients with Modic type 1 endplate lesions and patients with infectious spondylitis [13]. The authors found that the intra-vertebral PDFF, as well as the PDFF ratio of infectious spondylitis were statistically significantly lower compared to the corresponding values of patients with Modic type 1 endplate lesions, with areas under the curve (AUCs) of 0.977 for PDFF and 0.971 for the PDFF ratio, respectively [13]. Thus, lesions stemming from infectious spondylitis seem to be characterized by lower fat content than degenerative changes of Modic type 1 endplate lesions, potentially indicating that CSE-MRI-derived PDFF may qualify as a valuable quantitative imaging biomarker to facilitate the image-based differentiation between infectious and degenerative erosive changes at the vertebral endplates.

In addition to the fat quantification of vertebral BM, the CSE-MRI-derived PDFF has recently also been used to evaluate paraspinal muscle fat content [14,15,16]. For the degenerative spine, it has been shown that paraspinal muscle PDFF may be associated with lumbar back pain (LBP) and that cartilage endplate damage could be predictive of LBP when adjacent to paraspinal muscles with higher fat content [14,15]. Furthermore, anatomical variations in PDFF for paraspinal muscles have been explored in relation to gender and body mass index (BMI), demonstrating associations for PDFF values between segments of the erector spinae muscles at the lower thoracic and lumbar spine (T9-T11, T12-L2, and L3-L5) [16]. In an article of this Special Issue, Burian et al. expanded investigations to the cervical level and explored relationships between cervical and lumbar paraspinal muscle composition by means of the PDFF, as well as texture analysis (TA) applied to PDFF maps (cervical musculature and lumbar erector spinae and psoas muscles bilaterally) [17]. Healthy participants underwent CSE-MRI at the level of the cervical and lumbar spine at 3 Tesla, followed by paraspinal muscle segmentations with muscle-resolved PDFF and texture feature extraction [17]. The authors found a statistically significant difference between males and females for the PDFF of erector spinae, but not for psoas or cervical muscles [17]. The global texture features Kurtosis and Variance were statistically significantly higher in males than females for all segmented muscles [17]. Moreover, not only PDFF but also Variance, Entropy, Homogeneity, Correlation, and Energy showed statistically significant differences between the different segmented muscles [17]. Furthermore, Dieckmeyer et al. explored the role of paraspinal muscle PDFF and texture features from PDFF maps as predictors of muscle strength among 26 healthy participants at 3 Tesla by correlating these measures to muscle flexion and extension strength as measured by an isokinetic dynamometer [18]. They demonstrated that Kurtosis of the erector spinae muscles significantly correlated with extension strength (r = 0.59), while Variance in the psoas muscles was correlated to flexion strength (r = 0.63) [18]. Moreover, Kurtosis in the erector spinae muscles, as well as BMI, were statistically significant predictors of extension strength, while Skewness and Variance were statistically significant predictors of flexion strength (the PDFF alone was not identified as a statistically significant predictor) [18]. Taken together, paraspinal muscle fat content with derived TA could be used as a gender-specific measure with anatomical variation between muscle groups along the spine, which could have implications for body composition evaluation in sarcopenia or cachexia. Specifically, given the oftentimes limited coverage of the paraspinal musculature in clinical imaging (e.g., acquisition of images of the cervical, thoracic, or lumbar spine only according to clinical indication), knowledge about associations between body regions may help to estimate relationships between areas that are not fully covered by the imaging volume. Associations of certain texture features from PDFF maps with muscle strength may underscore crosslinks between image-based measures and muscle physiology and function.

Other articles in this Special Issue have set a focus on the opportunistic use of MDCT data for osteoporosis diagnostics or prediction of incidental osteoporotic VFs (i.e., use of existing imaging data originally acquired for other purposes than osteoporosis screening, such as oncologic staging in cancer patients) [19,20,21]. In detail, Burian et al. studied the contribution of bone mineral density (BMD) at different vertebral levels, subcutaneous adipose tissue (SAT), and visceral adipose tissue (VAT) regarding the identification of VFs using baseline and follow-up image data from a 64-row MDCT scanner in patients (osteoporotic incidental VFs) and controls (no VFs) [20]. The trabecular vertebral BMD (T5-L5), SAT, and VAT volumes were extracted from the imaging data [20]. It was revealed that the BMD performed best to differentiate patients with VFs from controls at the levels T5 (AUC = 0.781), T7 (AUC = 0.877), and T9 (AUC = 0.818), while, according to multivariate logistic regression (stepwise approach with baseline fracture status, BMD, SAT, VAT, and VAT/SAT ratio), only BMD at level T7 was a statistically significant predictor of VFs (T5-L5), with an odds ratio of 1.07 per BMD standard deviation decrease [20]. However, there was no statistically significant difference between patients and controls for VAT or SAT [20]. Furthermore, finite element analysis (FEA) as an advanced image analysis technique can be applied to MDCT data, which can relate morphological and material variations and properties to functional characteristics, thus providing 3D models of bone reconstructed from image data for the investigation of material properties within an FEA-meshed model [2]. In a study by Yeung et al., baseline routine MDCT scans (64-row MDCT scanner) of patients who sustained incidental osteoporotic VFs as confirmed in follow-up imaging were used for automated segmentation of thoracic and lumbar vertebrae (T5-L5), followed by BMD extraction and calculation of the FEA-based failure load and failure displacement [19]. The measurements of single vertebral bodies were normalized by dividing the absolute value by the average of L1–L3, as well as by dividing the absolute value by the average of T5–T12 and L1–L5, respectively [19]. The combination of normalized failure load, normalized failure displacement, and normalized BMD increased the AUC up to 0.77 [19]. Another study evaluated whether lumbar FEA can predict the biomechanical strength of functional spinal units (FSUs), which are given by at least two adjacent vertebrae with the intervertebral disc (IVD) [21]. Specifically, images acquired with a 64-row MDCT scanner among patients who sustained an incidental osteoporotic VF between baseline and follow-up exams were used to model two FSUs (L1–IVD–L2–IVD–L3 = FSU_L1–L3 and fractured vertebral body at the center of the FSU = FSU_F), followed by extraction of the BMD, the FEA-based displacement, and the FEA-based load [21]. The study revealed statistically significant correlations between the FSU_F and mean load of L1 to L3 (r = 0.814) and mean BMD of L1 to L3 (r = 0.745), adjusting for a BMD ratio of fracture/L1–L3 segments [21]. However, there were no statistically significant associations between the FSU_F and FSU_L1–L3 or between the FSU_F and the mean displacement of L1–L3 [21]. Taken together, the opportunistic use of spine MDCT data may have distinct value for fracture prediction, with FEA of single lumbar vertebrae having predictive capabilities regarding biomechanical strength assessments for incidentally fractured vertebral segments along the thoraco-lumbar spine. The combination of FEA with BMD may enable improved prediction of incidental osteoporotic VFs at vertebral-specific levels. Although relevant in conditions such as sarcopenia or cachexia, VAT and SAT may not be associated with incidental osteoporotic VFs on a clinically meaningful scale.

Two studies investigated radiological imaging after lumbar spinal instrumentation at the degenerative spine using radiography and structural MRI [22,23]. Angelini et al. analyzed the radiological outcome at the adjacent vertebral segment after lumbar stabilization with a hybrid stabilization system (with or without posterior lumbar inter-body fusion) in patients affected by degenerative lumbar disease (Modic score < 2 and Pfirrmann score < 4 and Weiner score < 2) [22]. Imaging was acquired prior to surgery as well as at one, six, and twelve months after surgery by means of radiographs (and MRI for the preoperative evaluation) among 27 consecutive patients [22]. The authors revealed statistically significant decreases in both the anterior and posterior disc height, as well as statistically significant changes in the segmental angle when comparing imaging over time, with eleven cases (40.7%) showing radiological progression of disc degeneration [22]. Hence, the authors concluded that the used hybrid stabilization system does not necessarily facilitate reduction in the progression of lumbar disc degeneration for neither the dynamized nor adjacent spinal segments, although such a hybrid stabilization system could establish a more harmonious biomechanical transition zone between the fused and adjacent segments. In a study by Byvaltsev et al., 80 patients who had undergone single-segment surgery (at L4/L5) were analyzed, and they were operated on with either open transpedicular screw fixation (TSF) or minimally invasive transforaminal interbody fusion (TLIF) with a rigid interspinous stabilizer, with unilateral TSF and contralateral facet fixation, or with bilateral TSF [23]. Evaluation of pre- and postoperative sagittal and axial T2-weighted sequences was performed using systematic grading by two readers, with an emphasis on image quality and extents of hardware-related artifacts [23]. Moderate to excellent inter-reader agreement was shown for scoring of artifacts in MRI data, with better image quality at the operated segment after minimally invasive procedures with a rigid interspinous stabilizer or with unilateral TSF and contralateral facet fixation as compared to deterioration for postoperative image quality following open TSF or bilateral TSF [23]. Furthermore, the area of the multifidus muscles indicated less atrophy over time for minimally invasive TLIF compared to open TLIF [23]. Hence, a minimally invasive approach may be considered beneficial in terms of avoiding marked postoperative artifacts in MRI data whilst reducing the extent of perioperative paraspinal muscle atrophy.

In a study by Janssen et al., a comparison of preoperative imaging findings from CT and MRI to intraoperative site inspection was performed for patients with suspected disco-ligamentous lesions of the subaxial cervical spine, with the objective of identifying radiological features for under- or over-estimating disco-ligamentous lesions [24]. Out of the included 83 patients, the majority underwent anterior cervical discectomy and fusion (ACDF; 79 patients), while the remaining patients underwent anterior cervical corpectomy and fusion (4 patients) [24]. A discrepancy between preoperative imaging and intraoperative surgical findings was revealed in 14 patients, with a specificity/sensitivity of preoperative imaging to identify disco-ligamentous lesions of 100%/77.4% [24]. When adding the presence of VFs and/or prevertebral hematoma to the model, an increased sensitivity of 95.2% was achieved [24]. Yet, the presence of marked degenerative changes at the cervical spine seems to represent a major risk factor for missing a traumatic disco-ligamentous injury during radiological image reading, and prevertebral hematoma and/or VFs are important image-based hints for co-existing disco-ligamentous injuries.

Regarding potential associations between the degenerative spine and BMD, Geng et al. investigated 745 participants (aged 20 to 60 years) by MRI to assess disc herniations and by dedicated quantitative CT (QCT) to extract BMD (L2–4) [25]. According to the results of their study, the differences in BMD between subjects without and those with single or ≥2 sites of lumbar disc herniation were not statistically significant, and no associations between BMD and lumbar disc herniation were observed in either men or women [25]. The degenerative spine was also in the focus of a study by Lehnen et al., who aimed to detect a variety of different degenerative changes of the lumbar spine (including presence of disc herniation, disc bulging, spinal canal stenosis, nerve root compression, and spondylolisthesis) by means of a convolutional neural network (CNN) trained on multiple MRI-based features [26]. Including T2-weighted imaging with labeling of vertebrae and IVD segments in 146 patients, the CNN-based algorithm’s diagnostic accuracy and consistency in relation to visual radiological image reading was evaluated, revealing perfect accuracy for IVD detection and labeling (100%) and moderate to high diagnostic accuracy for the detection of disc herniations (87%) or bulgings (76%) [26]. Furthermore, its accuracy for the detection of spinal canal stenosis (98%), nerve root compression (91%), and spondylolisthesis (87.6%) was also high [26]. These results suggest that evaluation of the degenerative spine with support by a CNN-based algorithm may automate image assessments, thus potentially limiting time expenses and efforts by radiologists. Amongst others, image data analyses of the degenerative spine supported by AI was also reflected on by a narrative review article of Mallio et al., who also reviewed and summarized advanced methods for imaging of IVD and cartilage degeneration [27]. Specifically, the authors included T1ρ and T2 relaxation mapping, T2* mapping, diffusion-weighted imaging, sodium MRI, glycosaminoglycan chemical exchange saturation transfer imaging, ultra-short echo time imaging, magnetic resonance spectroscopy, delayed Gadolinium-enhanced MRI, and imaging using the magnetization transfer ratio [27]. In addition to imaging for diagnostic purposes and clinical management, the authors pointed out that lumbar spine MRI could also serve as a tool for follow-up examinations after IVD regenerative therapy [27]. Once application of novel imaging acquisition and analysis tools has accomplished the transition to the clinical setting, it may facilitate early diagnosis and patient phenotyping, and it could guide conservative and regenerative treatments that may prevent the progression of degeneration along the spine.

In conclusion, the manuscripts published in this Special Issue provide new insights into state-of-the-art image acquisition techniques and analysis tools at the spine. However, the transfer of these findings to clinical practice remains challenging. We would like to thank the authors for their contributions and wish the readers happy and fruitful reading.
